# Machine-Learning-Based Prediction of Treatment Outcomes Using MR Imaging-Derived Quantitative Tumor Information in Patients with Sinonasal Squamous Cell Carcinomas: A Preliminary Study

**DOI:** 10.3390/cancers11060800

**Published:** 2019-06-10

**Authors:** Noriyuki Fujima, Yukie Shimizu, Daisuke Yoshida, Satoshi Kano, Takatsugu Mizumachi, Akihiro Homma, Koichi Yasuda, Rikiya Onimaru, Osamu Sakai, Kohsuke Kudo, Hiroki Shirato

**Affiliations:** 1 Department of Diagnostic and Interventional Radiology, Hokkaido University Hospital, Sapporo 060-8638, Hokkaido, Japan; yshimizu1222@yahoo.co.jp (Y.S.); panacea-hok@umin.net (D.Y.); kkudo@huhp.hokudai.ac.jp (K.K.); 2 Department of Otolaryngology-Head and Neck Surgery, Hokkaido University Graduate School of Medicine, Sapporo 060-8638, Hokkaido, Japan; skano@med.hokudai.ac.jp (S.K.); mizumati@med.hokudai.ac.jp (T.M.); ak-homma@med.hokudai.ac.jp (A.H.); 3 Department of Radiation Medicine, Hokkaido University Graduate School of Medicine, Sapporo 060-8638, Hokkaido, Japan; kyasuda@med.hokudai.ac.jp (K.Y.); ronimaru@med.hokudai.ac.jp (R.O.); shirato@med.hokudai.ac.jp (H.S.); 4 Departments of Radiology, Otolaryngology-Head and Neck Surgery, and Radiation Oncology, Boston Medical Center, Boston University School of Medicine, Boston, MA 02118, USA; Osamu.Sakai@bmc.org; 5 The Global Station for Quantum Medical Science and Engineering, Global Institution for Collaborative Research and Education, Sapporo 060-0808, Hokkaido, Japan

**Keywords:** magnetic resonance imaging, machine learning, diffusion, perfusion, texture analysis, squamous cell carcinoma of the head and neck

## Abstract

The purpose of this study was to determine the predictive power for treatment outcome of a machine-learning algorithm combining magnetic resonance imaging (MRI)-derived data in patients with sinonasal squamous cell carcinomas (SCCs). Thirty-six primary lesions in 36 patients were evaluated. Quantitative morphological parameters and intratumoral characteristics from T2-weighted images, tumor perfusion parameters from arterial spin labeling (ASL) and tumor diffusion parameters of five diffusion models from multi-b-value diffusion-weighted imaging (DWI) were obtained. Machine learning by a non-linear support vector machine (SVM) was used to construct the best diagnostic algorithm for the prediction of local control and failure. The diagnostic accuracy was evaluated using a 9-fold cross-validation scheme, dividing patients into training and validation sets. Classification criteria for the division of local control and failure in nine training sets could be constructed with a mean sensitivity of 0.98, specificity of 0.91, positive predictive value (PPV) of 0.94, negative predictive value (NPV) of 0.97, and accuracy of 0.96. The nine validation data sets showed a mean sensitivity of 1.0, specificity of 0.82, PPV of 0.86, NPV of 1.0, and accuracy of 0.92. In conclusion, a machine-learning algorithm using various MR imaging-derived data can be helpful for the prediction of treatment outcomes in patients with sinonasal SCCs.

## 1. Introduction

Multimodal treatment such as a combination of surgery, chemotherapy and radiotherapy is the standard treatment methods for head and neck squamous cell carcinomas (SCCs), including nasal cavity or paranasal sinus SCCs [1]. Superselective arterial infusion of cisplatin with concomitant radiotherapy has been widely recognized as a preferred treatment method for maxillary SCCs because of its high local control rate and good prognosis, especially in patients with advanced diseases [2]. In performing such nonsurgical therapy, the ability to predict treatment outcome, for instance whether local control will be achieved, is very important. High-accuracy data-based predictions based on individualized risk categories could help optimize management such as the selection of chemotherapy (e.g., decisions about whether to perform induction chemotherapy) and the best post-treatment follow-up strategy. In this way, truly personalized precision medicine may result from image-based data.

To assess and predict treatment outcomes, information about tumor, node, metastasis (TNM) staging and tumor size have been conventionally used. However, several studies have reported that such tumor conventional morphological information is not necessarily promising for predicting treatment outcomes [3,4]. In contrast, a wide variety of information can be obtained for any tumor from imaging such as quantitative tumor shape data [5], intratumoral textural parameters [6] and tumor functional information such as tumor tissue perfusion information from perfusion-weighted imaging [4,7] and tumor microstructural characteristics from diffusion-weighted imaging (DWI) [8,9,10]. Especially, several studies identified associations between diffusion parameters derived from DWI and various histopathological features, e.g. expression of Ki-67, p53, degree of tumor cellularity, etc. [11,12]. Tumor perfusion parameters derived from dynamic contrast enhanced (DCE) technique were also reported its correlation to tumor histopathological features such as p16 status and vascular endothelial growth factor (VEGF) expression [13]. Another report demonstrated even conventional magnetic resonance (MR) T1 weighted image (T1WI) and T2 weighted image (T2WI) showed the correlation to several histopathological features with the use of histogram image analysis [14]. In contrast, as another approach by different imaging modality, positron-emission tomography (PET) with ^18^F-fluorodeoxyglucose (FDG) depicts a tumor’s rate of glucose metabolism. Several reports indicated FDG-PET imaging parameters provided significant correlations to many histopathological features [15,16,17,18,19]. However, FDG-PET scanning was a little invasive because of its radiation exposure. In using MR imaging, most abovementioned tumor characteristics can be obtained completely non-invasively without the use of contrast agent and radiation exposure. Several of these MR derived parameters have been reported as independent predictors for treatment outcomes [4,8,20,21,22,23]. If we can efficiently integrate these data, prediction of the treatment outcome with high diagnostic accuracy may be obtained. 

Conventional multiparametric analysis is an important diagnostic method and will continue to provide clinically useful cut-off values for parameters. However, a more advanced analysis technique is needed to handle the variety of tumor characteristics that are now available. Recently, machine-learning algorithms have been reported to select various parameters effectively in medical imaging analysis including the assessment of head and neck SCC as a kind of artificial intelligence-based diagnostic technique [24,25], however, the number of reports is very limited and they have focused exclusively on intratumoral texture analysis parameters only; the utility of tumor functional information such as tumor perfusion and diffusion is still uncertain. The utilization of advanced parameters by machine learning may achieve ever-better predictive results by integrating data on numerous tumor characteristics.

The aim of this study was to determine the power of a machine-learning algorithm integrating multiple quantitative MR imaging data including shape parameters, intratumoral texture, perfusion and diffusion to predict treatment outcomes in patients with sinonasal SCCs. 

## 2. Results

Fourteen patients were revealed to have local failure. Of these 14 patients, local failure was proven in 11 by surgical biopsy with histological demonstration of residual SCC. In three patients, a large new mass lesion was observed during the follow-up period. The remaining 22 patients were determined to have local control by clinical observation throughout follow-up (mean 57 mos; range 34–86 mos). Imaging analyses of the primary sites were successfully performed in all patients. Details of obtained tumor morphological and intratumoral characteristics, perfusion and diffusion parameters are summarized in Table 1.

In the support vector machine (SVM) analysis, classification criteria for the discernment of local control versus failure in the training set data could be constructed with a sensitivity of 0.98, specificity of 0.91, positive predictive value of 0.94, negative predictive value of 0.97 and accuracy of 0.96, as a mean value obtained from the nine test set data. In the parameter selection process, the five top-ranked features in all nine training data sets included the perfusion parameter of relative tumor blood flow (TBF), morphological parameter of tumor sphericity, and texture parameter of contrast. In addition, the morphological parameter of tumor volume was selected six times, the diffusion parameter of intermediate diffusion coefficient (D_2_) in tri-exponential model DWI five times, the diffusion parameter of perfusion fraction f in the intravoxel incoherent motion (IVIM) DWI model three times, the texture parameter of energy twice, and both diffusion parameters of apparent diffusion coefficient (ADC) and patient characteristics of T-stage once. Details of results for training group data are presented in Table 2.

The classification algorithm for the discernment of local control versus failure obtained by training set data was further used for the classification in the validation set data. Group assignment in the validation data set was achieved with a sensitivity of 1.0, specificity of 0.82, positive predictive value of 0.86, negative predictive value of 1.0 and accuracy of 0.92; these values were the means of total 36 validation data (4 × 9 validation sets).

## 3. Discussion

Our findings revealed that a machine-learning-derived diagnostic algorithm by pre-treatment MR imaging-derived tumor data can highly predict the treatment outcome of local control versus failure in patients with sinonasal SCCs. To the best of our knowledge, the current study is the first report to indicate the utility of a machine learning-based diagnosis derived from quantitative morphological, intratumoral, perfusion and diffusion data. This diagnostic ability can be useful as a kind of artificial intelligence-based technique. Being able to predict the local outcome of the primary tumor would have a significant impact on treatment decision. If a poor outcome after chemoradiation therapy is strongly indicated, additional or alternate treatment plan such as induction chemotherapy, earlier salvage surgery after the chemoradiotherapy, or surgical resection prior to chemoradiation therapy may be offered. Follow-up strategy after the treatment such as the frequency and duration of the imaging follow-up may be also optimized individually, depending on the pre-treatment risks.

Quantitative morphological shape characteristics of tumors (i.e., sphericity) were recently reported to be useful for the prediction of treatment outcomes in head and neck SCC as an advanced morphological parameter compared to TNM stage and tumor size [5,23]. Intratumoral heterogeneity was also indicated as a prognostic factor in head and neck SCC patients [26,27]; in these reports, tumors with high degree of intratumoral heterogeneity demonstrated a tendency for poor prognoses. In contrast, tumor perfusion and diffusion are considered to be major tumor functional parameters that indirectly reflect biological processes; thus, these may be related to individual treatment sensitivity. In previous reports, tumor perfusion was described as an important predictor of the treatment outcome [4]. Lower tumor perfusion was related to poor treatment outcome in these reports, probably because of the relation between tumor hypoxia and lower tumor perfusion [28,29]. Tumor diffusion was also significantly related to treatment outcome in head and neck SCC patients; in numerous ADC studies [30], low ADC was related to a good treatment outcome. Additionally, non-Gaussian diffusion parameters beyond ADC values have been recently shown to be useful for treatment outcome prediction [8].

The various characteristics of tumor shape, intratumoral characteristics, and tumor perfusion and diffusion are expected to reflect different types of biological conditions. Multiparametric use of these data were suggested to allow each parameter’s diagnostic power to be expressed. A previous study used multiparametric analysis employing both ADC values and tumor perfusion information, and concluded that the combination of diffusion and perfusion data was helpful for diagnosis [31]. However, diagnostic performance was not still complete, probably because simple cut-offs are insufficient to fully reflect tumor biology. In the current study, the machine-learning algorithm seemed to emphasize all effective parameters sufficiently. The high diagnostic accuracy of treatment outcome prediction obtained by the present study is probably due to the effective use of numerous tumor characteristics with an ideal determination strategy. The various characteristics differ from patient to patient; non-invasive acquisition of numerous tumor characteristics using advanced MR imaging sequence and post-processing techniques and determination of the diagnostic strategy using this machine-learning algorithm should contribute greatly to individualized precision medicine for sinonasal SCC patients.

The current study has several limitations. First, the number of patients was quite small. However, sinonasal SCC are not very common, and it would be a challenge to investigate a larger number of sinonasal SCC patients who received advanced MR imaging protocol and long-term follow-up. The results of the present study should be regarded as preliminary. Second, analysis of intratumoral characteristics was performed only in T2WI. Arterial spin labeling (ASL) and DWI were acquired with the use of large pixel size to obtain sufficient signal-to-noise ratios; therefore, especially complex intratumoral analysis such as second-order texture was somewhat difficult. Additionally, post-contrast-enhanced T1WI is somewhat invasive, and differences in readout schemes such as 2D or 3D acquisition affect intratumoral texture data. Third, although a few studies which performed radiomics approach to predict treatment outcome in patients with head and neck cancer were recently reported [32,33,34,35], whether diagnostic power obtained in the current study will be superior or not compared to previous reports is still unclear. It was because patient characteristics, treatment method, follow-up strategy, imaging technique and diagnostic model construction algorism were quite different among these studies. Further analysis to address these limitations will be needed.

## 4. Materials and Methods

### 4.1. Subjects

This retrospective study was approved by the institutional review board of Hokkaido University Hospital (ID: 017-0322, date of approval: 28th February 2018), and the requirement to obtain written informed consent was waived. From September 2010 to October 2015, 36 consecutive patients with histopathologically proven sinonasal SCCs who were referred to our hospital to undergo superselective arterial infusion of cisplatin with concomitant radiotherapy were enrolled in this study. Patient characteristics are summarized in Table 3.

The treatment regimen was a superselective arterial infusion of cisplatin with concomitant radiotherapy for all patients. Superselective transarterial infusion therapy with cisplatin (100–120 mg/m^2^ per week for 4 weeks) via the dominant feeding arteries to the primary tumor was performed using a microcatheter, with concurrent radiotherapy of a total of 70 Gy in 35 fractions with curative intent.

### 4.2. MR Imaging Protocol

All MR imaging was performed using a 3.0-Tesla unit (Achieva TX; Philips Healthcare, Best, The Netherlands) with a 16-channel neurovascular coil. MR imaging including conventional anatomical imaging of spin-echo (SE) T1WIs, turbo spin-echo (TSE) fat-suppressed (Fs) T2WIs, ASL and multiple b-value DWI was performed before treatment in all patients. The patients were instructed not to swallow, move their tongues, open their mouths, or make any other voluntary motion during the MR scan. All axial slices were placed in parallel with the anterior commissure-posterior commissure line.

#### 4.2.1. Conventional MR Imaging Acquisition

Conventional MR imaging sequences were obtained to evaluate the primary tumor including axial SE T1WIs (TR, 450 ms; TE, 10 ms; field of view (FOV), 240 × 240 mm; 512 × 512 matrix; slice thickness, 5 mm; inter-slice gap, 30%; scanning time, 2 min 12 s), and axial TSE Fs-T2WIs (TR, 4500 ms; TE, 70 ms; TSE factor, 9; FOV, 240 × 240 mm; 512 × 512 matrix; slice thickness, 5 mm; inter-slice gap, 30%; scanning time, 2 min 06 s). Coronal TSE T2WIs (TR, 4,500 ms; TE, 70 ms; TSE factor, 9; FOV, 240 × 240 mm; matrix, 512 × 512; slice thickness, 4 mm; inter-slice gap, 30%; scanning time, 2 min 06 s) were also obtained as a supporting tool for ASL acquisition (*see below*).

#### 4.2.2. ASL Acquisition

For the ASL imaging, a pseudo-continuous ASL (pCASL) technique was used. The acquisition of pCASL was performed using multi-shot spin-echo echo-planar imaging to obtain control and labeled images. The labeling slab was placed just under the bifurcation of the internal and external carotid arteries using coronal T2WIs as a reference for the placement of the labeling plane by carefully detecting the vessel flow void. Control images were obtained without the labeling of arterial water, using the same imaging scheme as the labeled images. The acquisition parameters of the pCASL were as follows: labeling duration, 1650 ms; post-label delay, 1280 ms; TR, 3619 ms; TE, 18 ms; flip angle, 90°; number of shots, two; FOV, 230 × 230 mm; matrix, 80 × 80; slice thickness, 5 mm; number of slices, 15; acceleration factor for parallel imaging, two; scanning time, 5 min 11 s. ASL-based perfusion-weighted images were obtained by subtracting values of the control from the labeled images.

#### 4.2.3. DWI Acquisition

The DWI acquisition used single-shot spin-echo echo-planar imaging (EPI) with three orthogonal motion-probing gradients. Twelve b-values (0, 10, 20, 30, 50, 80, 100, 200, 400, 800, 1,000, and 2000 s/mm^2^) were used. Other imaging parameters were: TR, 4500 ms; TE, 64 ms; DELTA (large delta; gradient time interval), 30.1 ms; delta (small delta; gradient duration), 24.3 ms; flip angle, 90°; FOV, 230 × 230 mm; 64 × 64 matrix; slice thickness, 5 mm × 20 slices; voxel size 3.59 × 3.59 × 5.00 mm; parallel imaging acceleration factor, 2; numbers of signal averages, b-value of 0–100 s/mm^2^ (one average), 200–800 s/mm^2^ (two averages) and 1000–2000 s/mm^2^ (three averages); scanning time, 4 min 37 s.

### 4.3. Data Analysis

#### 4.3.1. ROI Delineation

A board-certified neuroradiologist with ten years’ experience in head and neck imaging delineated each tumor with a polygonal region-of-interest (ROI) on Fs-T2WIs, ASL subtraction images and DWI b0 images. The ROI of the axial Fs-T2WI was placed first; then almost the same ROI was delineated with reference to the Fs-T2WI ROI so that the same region was delineated for all sequences. For the DWI analysis, the tumor ROI on the b0 image was copied on the echo planar imaging (EPI) of the respective b-values. Any area suspected of being a necrotic or a cystic lesion was excluded from the ROI. If the tumor extended into two or more slices, all slices were delineated respectively. In addition, the medial pterygoid muscle of the healthy side was also delineated with a polygonal ROI on Fs-T2WI and ASL images for the relative T2WI signal intensity and perfusion parameter calculation with reference to the axial Fs-T2WI.

#### 4.3.2. Analysis of Fs-T2WI for Morphological and Intratumoral Data

For the analysis of morphological characteristics, we calculated the tumor volume and tumor surface area from the morphological shape of all selected tumor voxels with the delineated tumor ROI on Fs-T2WI. The sphericity of each tumor was calculated by a previously reported method [23]. For the intra-tumoral characteristics analysis, we calculated the relative Fs-T2WI mean signal intensity (=mean signal on tumor ROI/ the medial pterygoid muscle ROI), the coefficient of variance (CV) in the tumor ROI and the textural parameters within the ROI. Textural features included the contrast, correlation, energy, and homogeneity, based on gray-level co-occurrence matrix (GLCM) features, namely, spatially detailed signal intensity data within the tumor ROI; GLCM were previously described the most common and sensitive texture descriptor to calculate lesion heterogeneity in greater detail from the texture data [36]. Texture parameters were calculated based on a previously reported method [37].

#### 4.3.3. ASL and DWI Analysis

We calculated the TBF of the pCASL from the signal data in ASL subtraction images using the previously described equation [7]. Both absolute and relative TBF values were calculated, with absolute TBF being the mean TBF value in the delineated tumor ROI, and relative TBF being that value divided by the medial pterygoid muscle blood flow, i.e., the mean value in the muscle ROI [7]. From the diffusion signal data, we calculated each parameter of mono-exponential function (i.e., ADC), bi-exponential function so-called IVIM (the perfusion fraction f, the pseudo-diffusion coefficient D*, and the true diffusion coefficient D), the tri-exponential function (the perfusion-related diffusion fraction f_1_ and coefficient D_1_, the intermediate diffusion fraction f_2_ and coefficient D_2_, and the slow diffusion fraction f_3_ and coefficient D_3_), the stretched exponential model (SEM) (diffusion heterogeneity α and distributed diffusion coefficient DDC) and the diffusion kurtosis imaging (DKI) (kurtosis value K and kurtosis-corrected diffusion coefficient D_k_). Using the signal intensities of all 12 b-values, we calculated the bi-exponential and tri-exponential function parameters. Assessments of the ADC, SEM and DKI usually target tissue diffusion (except for the perfusion-related signal), and we therefore used the signal intensity of six b-values (0, 200, 400, 800, 1000 and 2000 s/mm^2^) for the parameter calculations of ADC, DKI and SEM. To perform these parameter calculations, we used the previously reported calculation method [38]. In-house developed program by MATLAB ver. 2015a (MathWorks, Natick, MA, USA) was used for the above-described calculations. 

### 4.4. Determination of the Clinical Outcome

In all patients, clinical and radiological follow-up was performed for ≥ 2 years after treatment to determine the final diagnosis of local control/failure at the primary site. During follow-up period, if residual/recurrent tumor was suspected by visual inspection or radiological assessment (e.g., development of mass like lesion in post-treatment granulation tissue), surgical resection was performed to confirm the presence of histological residual/recurrent tumor. If patient’s consent for the surgical resection was not obtained, careful observation was continued. Local failure was defined by the histopathological confirmation of SCC in biopsied or surgically resected tissue, or the observation of clearly development/enlargement of a mass lesion arisen from post-treatment granulation. Local control was defined by histopathological confirmation of the absence of SCC by surgical resection and no enlargement of soft tissue in post-treatment granulation throughout the follow-up period. 

### 4.5. Statistical Analysis Based on Machine Learning Algorithm

**Figure 1:**
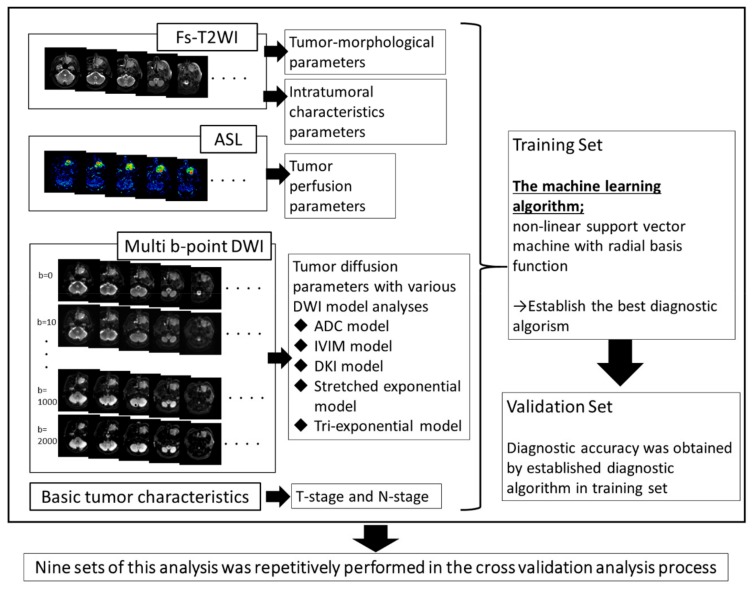
The overall process for parameter acquisition and machine-learning analysis.

A machine-learning algorithm by non-linear SVM with radial basis function was used to obtain the best diagnostic algorithm for the differentiation of local control versus failure groups. The kernel size (γ) and regularization parameter (C) with the best performance to predict the local control versus failure group was determined by the grid-search technique within ranges of 2^−20^ to 2^10^ for γ and from 2^−10^ to 2^10^ for C. In the feature-selection process, we performed backward sequential selection to determine the ranking in all features as well as the best diagnostic algorithm. This method is based on an iterative technique in which the starting condition includes all features. With each iteration, the feature with the least impact on improving the diagnostic performance for the differentiation of local control versus failure is eliminated. Features are removed one by one, and the rank of each feature is determined by the number of the iteration in which it was eliminated. Higher ranked features are explained that remained through the longest number of iterations. Ultimately, several highly ranked features that showed the highest diagnostic accuracy for predicting local control versus failure groups were included in the final established diagnostic model [39]. To avoid the possibility of overfitting with the use of leave-one-out cross-validation technique, the accuracy of the prediction was evaluated through a 9-fold cross-validation scheme. The data were randomly divided into 9 equal-size subsets (i.e. four patients each), of which eight subsets of the 32 (4 × 8) patients were used for training and the remaining one of four (4 × 1) patients for validating. The mean diagnostic accuracy over 9 repetitions was calculated for all possible combinations of features to find the combination of features with the highest predictive power. The overall analytic process is summarized in Figure 1. All of the analysis was performed using MATLAB ver. 2015a.

Morphological and intratumoral data from Fs-T2WI, perfusion data from ASL and diffusion data from DWI were respectively obtained by post-processing image analysis. The diagnostic algorithm was constructed by the machine-learning technique from all the obtained parameters in the training set patient group; then, the diagnostic accuracy of the constructed algorithm was calculated from the data in the validation set. This process was repetitively performed 9 times in each set of training and validation groups in a total cross-validation analysis.

## 5. Conclusions

The integration of quantitative tumor morphological, intratumoral textural, perfusion and diffusion data with the machine-learning-based analysis might be highly accurate at predicting treatment outcomes in patients with sinonasal SCC. This information could help in making decisions regarding treatment options such as details of treatment planning, additional treatment, and follow-up strategies, and thus to improved patient prognoses.

## Figures and Tables

**Table 1:** Data of patients with local control and failure.

			Treatment Outcome	
			Local Control	Local Failure
**Patients’ Characteristics (no. of patients)**	T-stage	T1	0	0
		T2	1	0
		T3	10	3
		T4	11	11
	N-stage	N0	18	12
		N1	2	0
		N2	2	2
		N3	0	0
**Morphological Parameters**	Tumor volume (mL)		25.3 ± 16.5	34.3 ± 28.6
	Surface area (cm^2^)		50.4 ± 18.7	69.6 ± 29.7
	Sphericity		0.71 ± 0.08	0.61 ± 0.11
**Intratumoral Characteristics Parameters**	Relative mean signal		3.8 ± 0.6	3.4 ± 0.6
	Coefficient of variance		0.12 ± 0.02	0.14 ± 0.04
	Contrast		35.1 ± 6	41.3 ± 8.6
	Correlation		0.84 ± 0.02	0.86 ± 0.03
	Energy (×10^−3^)		1.5 ± 0.3	1.2 ± 0.4
	Homogeneity		0.28 ± 0.03	0.26 ± 0.03
**Perfusion Parameters**	Absolute TBF (mL/100g/min)		156.7 ± 32.9	133.7 ± 29.3
	Relative TBF		7.47 ± 0.83	6.25 ± 1.22
**Diffusion Parameters**	ADC (×10^−3^ mm^2^/s)		0.91 ± 0.1	0.87 ± 0.13
	f (×10^2^ %)		0.16 ± 0.05	0.16 ± 0.07
	D* (×10^−3^ mm^2^/s)		19.5 ± 7.5	16.7 ± 5.7
	D (×10^−3^ mm^2^/s)		0.75 ± 0.06	0.73 ± 0.09
	K		0.73 ± 0.07	0.76 ± 0.08
	D_k_ (×10^−3^ mm^2^/s)		1.24 ± 0.14	1.22 ± 0.19
	alpha (α)		0.69 ± 0.07	0.67 ± 0.08
	DDC (×10^−3^ mm^2^/s)		1.14 ± 0.12	1.12 ± 0.17
	f_1_ (×10^2^ %)		0.14 ± 0.04	0.13 ± 0.04
	f_2_ (×10^2^ %)		0.23 ± 0.04	0.25 ± 0.05
	f_3_ (×10^2^ %)		0.62 ± 0.06	0.61 ± 0.08
	D_1_ (×10^−3^ mm^2^/s)		32.9 ± 7.8	28.1 ± 6.5
	D_2_ (×10^−3^ mm^2^/s)		1.03 ± 0.16	0.92 ± 0.15
	D_3_ (×10^−3^ mm^2^/s)		0.64 ± 0.07	0.62 ± 0.1

Data are means ± standard deviations. TBF, tumor blood flow; ADC, apparent diffusion coefficient; f, perfusion fraction; D*, fast diffusion coefficient; D, true diffusion coefficient; K, kurtosis value; D_k_, kurtosis corrected diffusion coefficient; α, diffusion heterogeneity; DDC, distributed diffusion coefficient; f_1_, perfusion-related diffusion fraction; f_2_, intermediate diffusion fraction; f_3_, slow diffusion fraction; D_1_, perfusion-related diffusion coefficient; D_2_, intermediate diffusion coefficient; D_3_, slow diffusion coefficient.

**Table 2:** Results of the Training Set Data (n=32 in each set).

Set No.	Sensitivity	Specificity	PPV	NPV	Accuracy
1	1	0.92	0.95	1	0.97
Top 5 ranked variables: Sphericity, Relative TBF, Contrast, D2, f					
2	1	1	1	1	1
Top 5 ranked variables: Relative TBF, Sphericity, Contrast, T-stage, Tumor volume					
3	1	0.85	0.9	1	0.94
Top 5 ranked variables: Relative TBF, Contrast, Sphericity, Tumor volume, f					
4	0.95	0.78	0.91	0.9	0.91
Top 5 ranked variables: Relative TBF, Sphericity, D2, Energy, Contrast					
5	1	1	1	1	1
Top 5 ranked variables: Sphericity, Relative TBF, D2, Contrast, Tumor volume					
6	1	0.85	0.9	1	0.94
Top 5 ranked variables: Relative TBF, Sphericity, f, Contrast, Energy					
7	1	0.92	0.95	1	0.97
Top 5 ranked variables: Sphericity, Relative TBF, Contrast, Tumor volume, ADC					
8	0.94	0.93	0.94	0.93	0.94
Top 5 ranked variables: Relative TBF, Sphericity, Contrast, D2, Tumor volume					
9	0.95	0.9	0.95	0.9	0.94
Top 5 ranked variables: Relative TBF, Sphericity, D2, Contrast, Tumor volume					
**Average**	**0.98**	**0.91**	**0.94**	**0.97**	**0.96**

PPV, positive predictive value; NPV, negative predictive value; TBF, tumor blood flow; D_2_, intermediate diffusion coefficient; f, perfusion fraction; ADC, apparent diffusion coefficient.

**Table 3:** Patient characteristics (n = 36).

	Number of Patients
Age	
Range	43–73
Median	59
Average	58.7
Gender	
Male	28
Female	8
Primary tumor site	
Nasal cavity	6
Paranasal sinus	30
T-stage	
T1	0
T2	1
T3	13
T4a	17
T4b	5
N-stage	
N0	30
N1	2
N2	4
N3	0
Smoking status	
Tabacco smokers	31
Packs-years	
Range	2–161
Median	34
Average	40.4
Alcohol use	
Occasional or non-drinker	10
Moderate use	6
Heavy use	20
